# London Journal of Primary Care – a PAUSE

**DOI:** 10.1080/17571472.2018.1465636

**Published:** 2018-05-06

**Authors:** Paul Thomas

This is the penultimate Issue of LJPC. In August, we will PAUSE to consider a new stage.

LJPC started in 2008, the year of the 60th anniversary of the UK National Health Service (NHS) and the 30th anniversary of the World Health Organisation (WHO) Alma Ata Declaration. Both the NHS and WHO advocate whole system collaboration for a healthy society. This means much more than medical care. It means integrating multiple efforts to build local health communities, including self-care, shared-care and health promotion, as well as curative medicine. How to practically do this is the question that has dominated LJPC thinking ever since.

In the last 10 years, LJPC has published over 300 papers about how to achieve the WHO/NHS vision of community-oriented integrated care and health promotion. Papers have evaluated systems of care and described what health means in complex situations. John Horder, one of the most visionary general practitioners of all time, serialised his autobiography in LJPC. In particular, LJPC has co-produced papers, bringing together many different perspectives to explore *wicked problems*.

London Journal of Primary Care has now outgrown its origins. It has expanded beyond London to become an international, peer-reviewed, PubMed-cited journal. It is a social movement more than a journal. It is concerned with whole society health as well as primary care. So we have chosen to close the journal in its present form and offer what we have learned to others who might want to lead a next stage(s). We think of it as a PAUSE. The LJPC website will remain and the papers will also be available on the RCGP website as well as on PubMed.

This Issue reveals the breadth of LJPC interests. It includes:

John Ashton’s Lecture at Manchester Medical Society on 31st January 2018 reminds us that the NHS was never meant to be the only thing to improve the health of the population, but ‘one part only of an attack upon five giant evils: Physical Want, Disease, Ignorance, Squalor, and Idleness’. [1].

John reminds us that the Alma Ata Declaration of 1978 agrees with NHS principles. It underpins WHO strategy for health care

Health Services grounded in a whole population, whole system approach, the reorientation of health care towards primary and community care and upstream to prevention, tackling inequalities in health, full public engagement and partnership working and policies that support health within supportive environments.

Yet this is not happening enough. John argues that to change this we need ‘a vision of a whole system that tackles the 5 Giants, is rooted in public health and strong primary care, that has a convincing narrative, authentic leadership, full public engagement and adequate resources’.

Canada is one country that has done more than most to align public health and primary care. Quebec is the second most populous province in Canada. In 2004, Quebec developed Local Health Networks to facilitate better collaboration at the local level [2]. A paper in this Issue of LJPC discusses the impact of the 2015 health care reforms that merged public health care organisations under a single governance [3]. Some argue that these larger organisations would better manage joint budgets and achieve economies of scale. Others argue that centralisation of decision-making powers disempowers grassroots practice. The authors maintain that the mechanisms to achieve community-oriented integrated care need to be understood and evaluated on their ability to engage all health and social care providers, as well as physicians. Then their strengths and weaknesses can be better understood.

In the UK, the latest policy to integrate health and social care services at local level is called the ‘Five Year Forward View’ (5YFV). It gave rise to ‘New Care Models’ with strong general practitioner (GP) leadership. Humphrey and Cleaver gathered the views of 10 GPs from three clinical commissioning groups in South East England about the 5YFV [4]. The respondents were largely supportive of the underlying principles, but were concerned at the volume of work, increased expectations and lack of adequate resources. They considered that GPs of the future would require high-level communication and negotiation skills, entrepreneurial flair and skills in bid writing and tendering.

Three other papers in this Issue of LJPC describe innovations that reveal something of grassroots thinking about how to practically achieve community-oriented integrated care and health promotion.

Crocombe reviewed 249 discharge letters from secondary care and out of hours care to a large suburban general practice in East London that had been delayed by 18–24 months [5]. They found no adverse outcomes by the delay to patient care or safety. The authors advocate a transition away from paper communications towards fully integrated electronic care records and communication systems between primary care and other NHS services.

Meehan interviewed 30 patients about their experience of the ‘Discharge to Assess’ scheme. This was intended to avoid delayed hospital discharge by assessing need for care and support in the patient’s own familiar environment [6]. Sixty per cent of patients positively rated the scheme. Forty per cent of patients and 35% of carers felt that they had not been adequately consulted about decisions being made.

Abel describes the value of a peer support group for people who have been bereaved [7]. Participants mainly came from a pre-bereavement course that had helped them build friendships with others before the death happened.

We may not have achieved community-oriented integrated care, but LJPC papers of the past 10 years evidence a flowering of understanding of how to achieve it in the future.

Paul Thomas *LJPC Editor* Paul.thomas7@nhs.net
